# Supplementary material for: COSIGT: population-scalable genotyping of complex loci from low-coverage sequencing data using pangenome graphs
Source: Genome Biol. 2026 Sep 8;27:286. doi: 10.1186/s13059-026-04242-4 (PMC13551797; doi:10.1186/s13059-026-04242-4)
Supplement: Supplementary file 1 [file 13059_2026_4242_MOESM1_ESM.pdf]

Additional file 1

This file contains supplementary figures detailing the COSIGT pipeline workflows, computational resource usage, and comprehensive benchmarking results (across modern CMRGs, SVs, and simulated aDNA), along with supplementary data notes providing guidelines for reference preparation.

Table of Contents

A. Supplementary Figures

- **Fig. S1–S3:** Directed acyclic graphs (DAGs) of the COSIGT pipeline branches (`master`, `custom_alleles`, `ancient_dna`).
- **Fig. S4–S8:** Gene-level performance in leave-zero-out analysis for CMRGs (1X, 2X, 5X, 30X) and SVs (30X).
- **Fig. S9–S11:** Gene-level performance in leave-all-out analysis for CMRGs, SVs, and ancestry-mismatched CMRGs.
- **Fig. S12–S13:** Gene-level performance on simulated ancient DNA (aDNA) under varying coverage, contamination, and demographic scenarios.
- **Fig. S14–S17:** COSIGT performance in leave-zero-out analyses stratified by region length, region complexity, zygosity, and SV type.
- **Fig. S18–S19:** COSIGT performance on HLA typing compared to microarray-based imputation.
- **Fig. S20:** Empirical threshold for filtering low-confidence COSIGT genotype calls.
- **Fig. S21–S22:** COSIGT computational resource profiling (runtime and memory usage).

B. Supplementary Data Notes

- **Note S1:** Chromosome partitioning of haplotype-resolved assemblies.
- **Note S2:** Incorporating alternative reference contigs for target regions.

Supplementary Figures

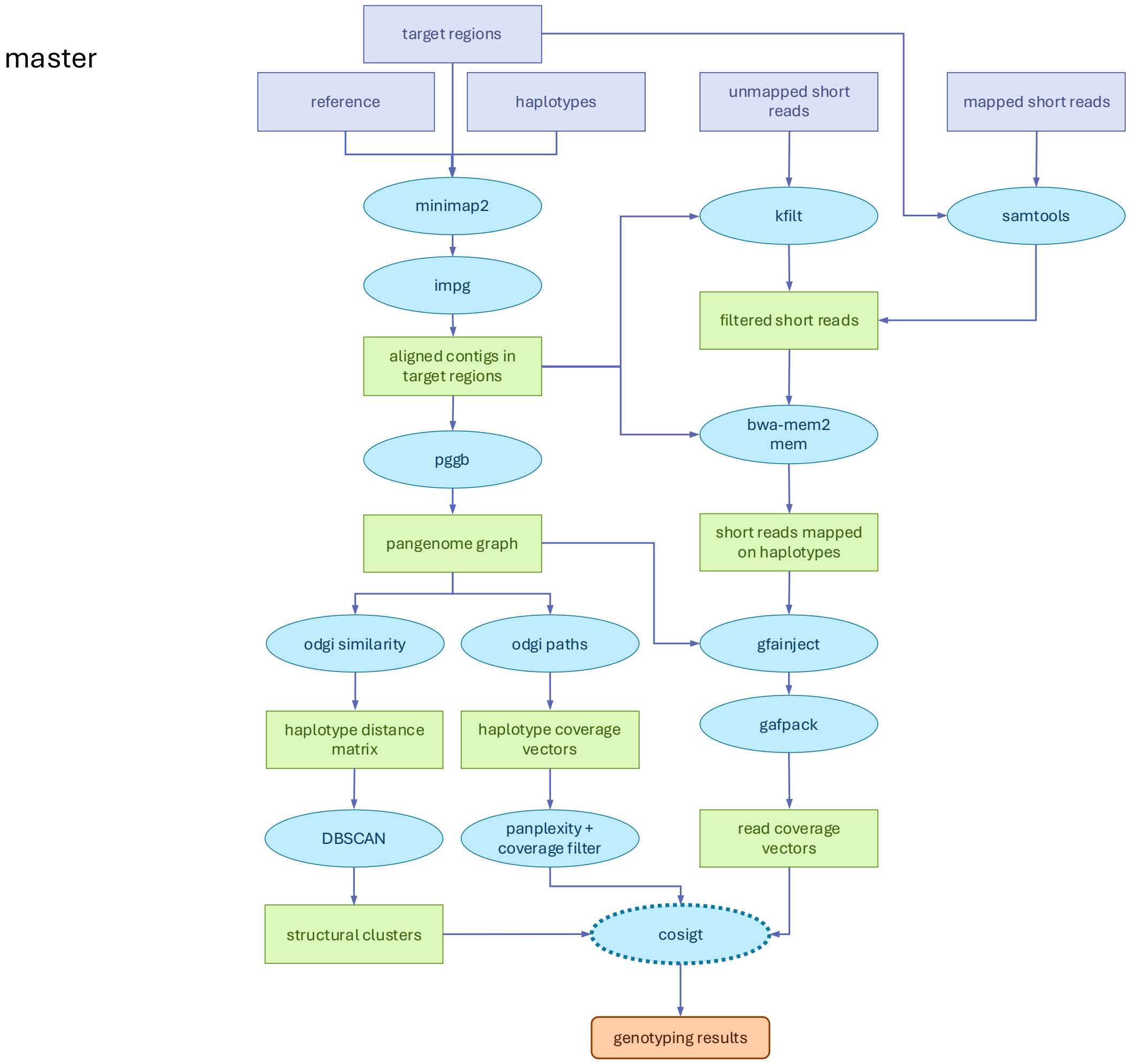

Fig. S1: **DAG of the COSIGT `master` pipeline branch.** Simplified directed acyclic graph (DAG) describing the `master` branch of the COSIGT pipeline, for the `cosigt` subcommand. Only required inputs and main outputs are represented. Purple rectangles: required input files. Blue ovals: main tools utilized by the pipeline. Green rectangles: main intermediate results. Orange rounded rectangle: final genotyping results. See Methods.

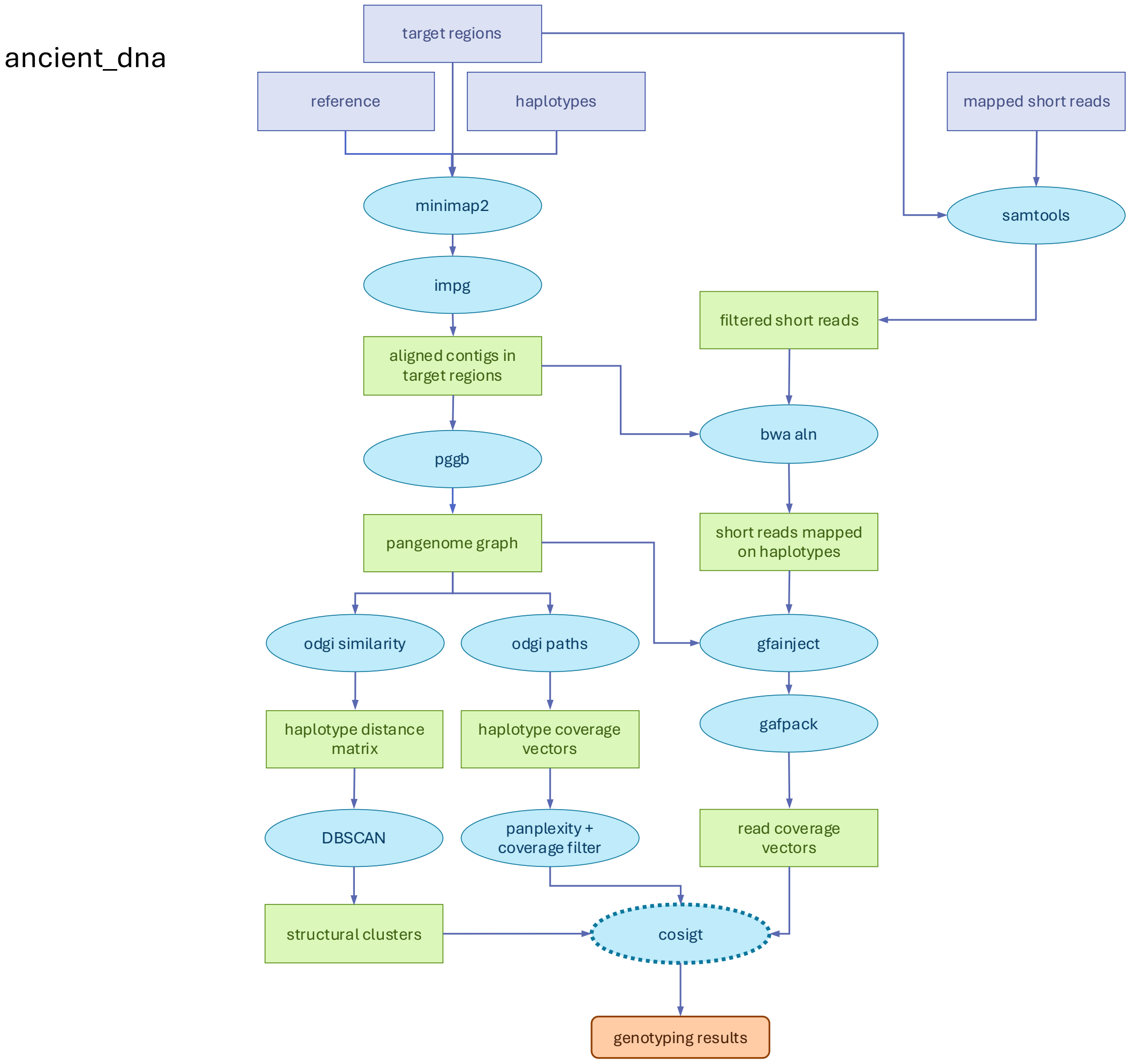

Fig. S2: **DAG of the COSIGT `custom_alleles` pipeline branch.** Simplified DAG describing the `custom_alleles` branch of the COSIGT pipeline for the `cosigt` subcommand. Compared to the `master` branch, `custom_alleles` skips the alignment of contigs to the target regions and exploits user-provided alleles for graph building and read alignment. See Methods.

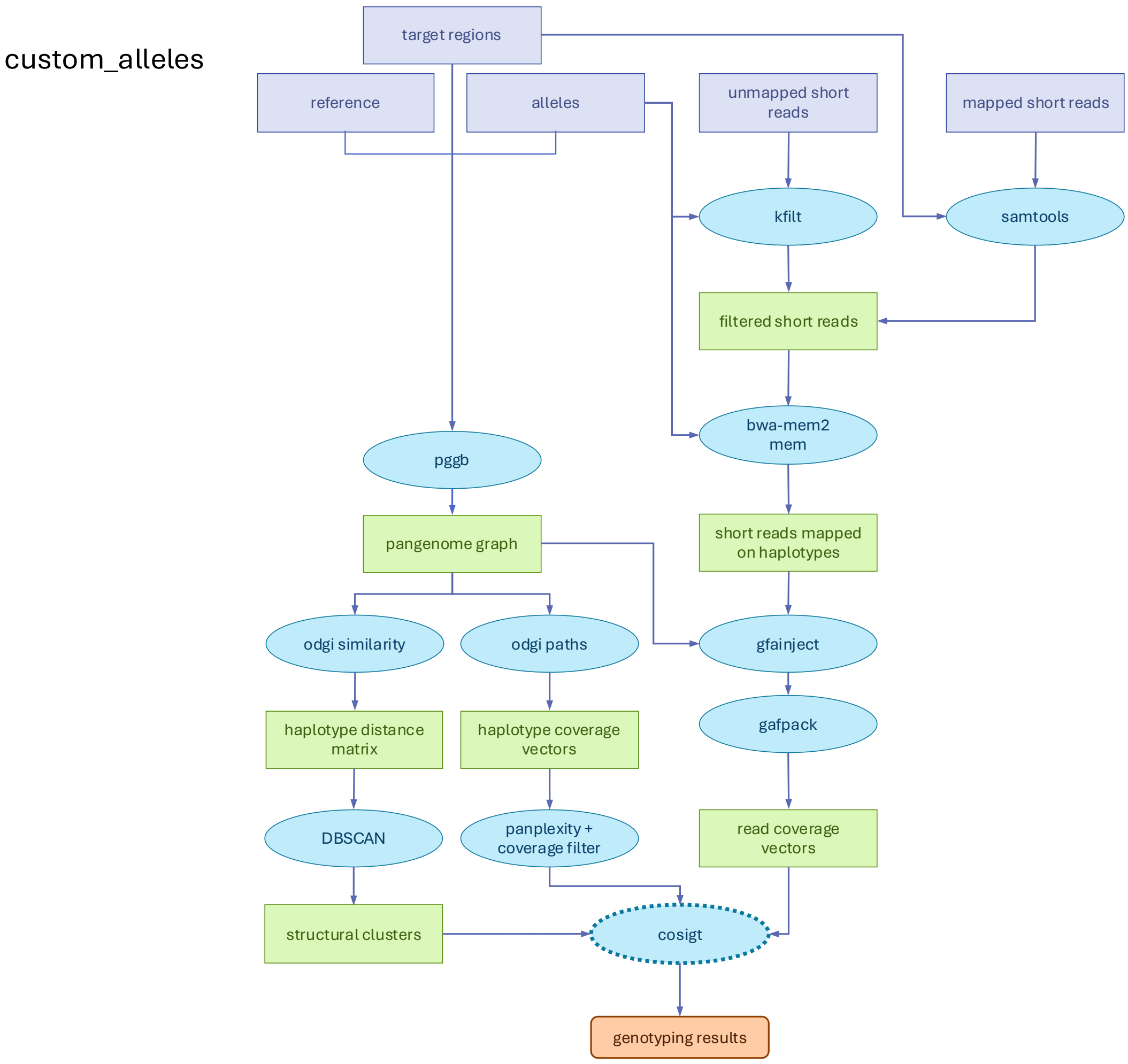

Fig. S3: **DAG of the COSIGT `ancient_dna` pipeline branch.** Simplified DAG describing the `ancient_dna` branch of the COSIGT pipeline for the `cosigt` subcommand. Compared to the `master` branch, this branch skips the recruitment of unmapped reads and replaces bwa-mem2 `mem` algorithm with bwa `aln` for read-to-haplotype alignment. See Methods.

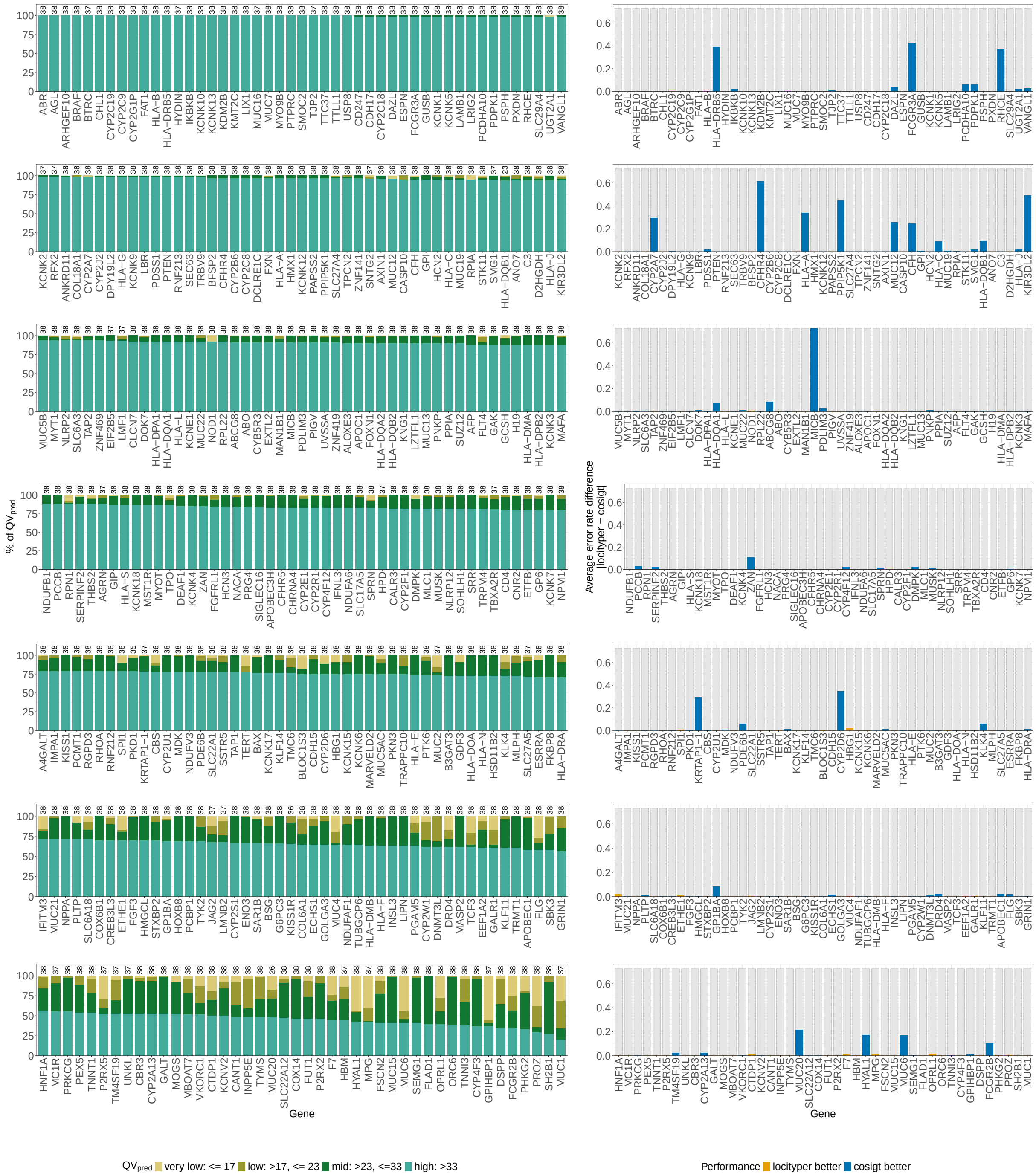

Fig. S4: **Gene-level COSIGT performance in leave-zero-out analysis of CMRGs at 1X coverage.** Per-gene comparison of COSIGT and Locityper genotyping accuracy for 326 CMRGs at 1X coverage. Left: QV_{pred} distribution for COSIGT, showing the percentage of samples in each quality category (very low: <17, low: 17–23, mid: 23–33, high: >33; see Methods). Sample counts per gene are indicated above each bar. Right: Absolute error rate differences between COSIGT and Locityper. Genes are ordered by COSIGT performance (higher to lower percentage of high QV calls per gene).

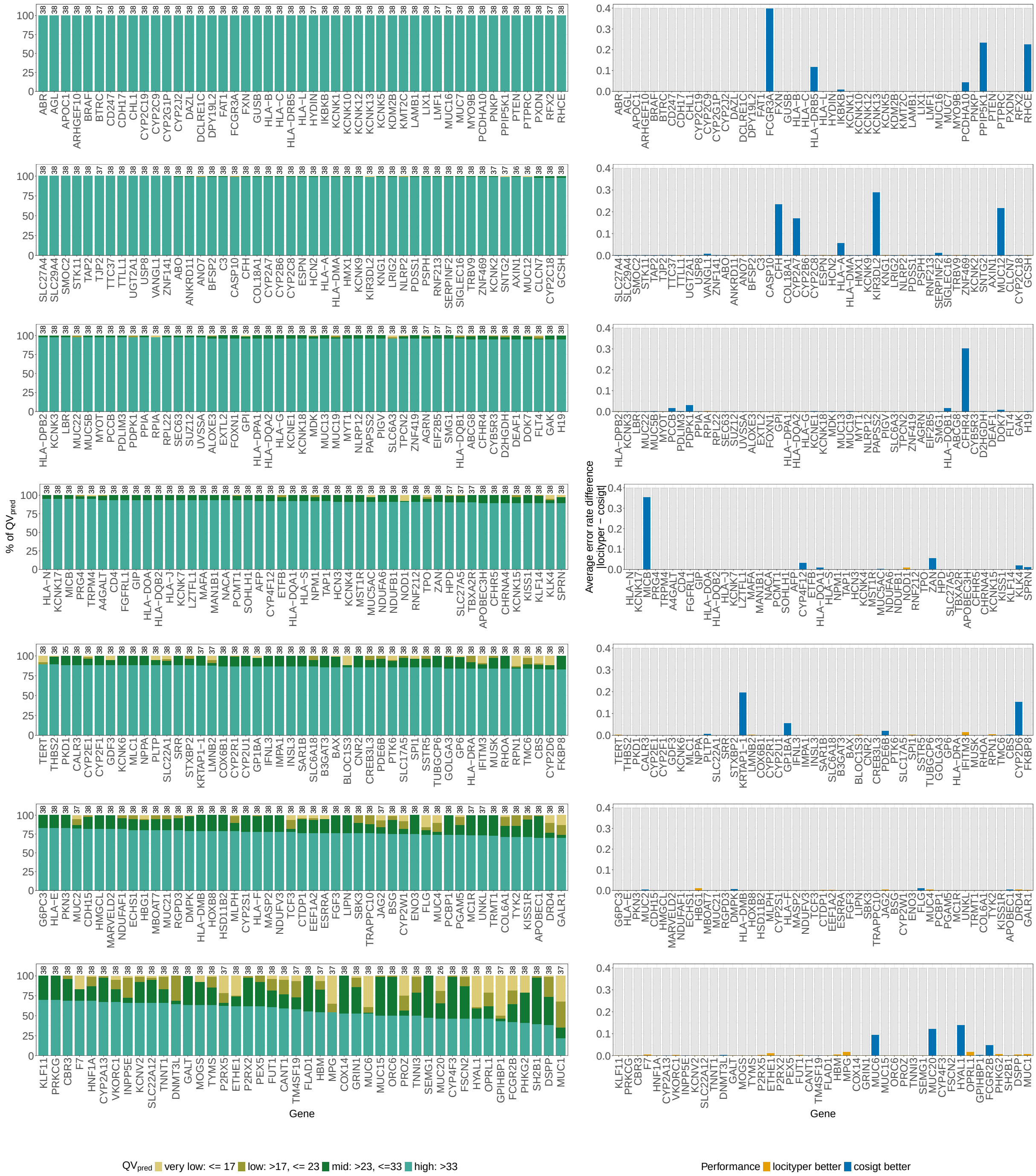

Fig. S5: **Gene-level COSIGT performance in leave-zero-out analysis of CMRGs at 2X coverage.** Per-gene comparison of COSIGT and Locityper genotyping accuracy for 326 CMRGs at 2X coverage. Left: QV_{pred} distribution for COSIGT, showing the percentage of samples in each quality category (very low: <17, low: 17–23, mid: 23–33, high: >33; see Methods). Sample counts per gene are indicated above each bar. Right: Average absolute error rate differences between COSIGT and Locityper. Genes are ordered by COSIGT performance (higher to lower percentage of high QV calls per gene).

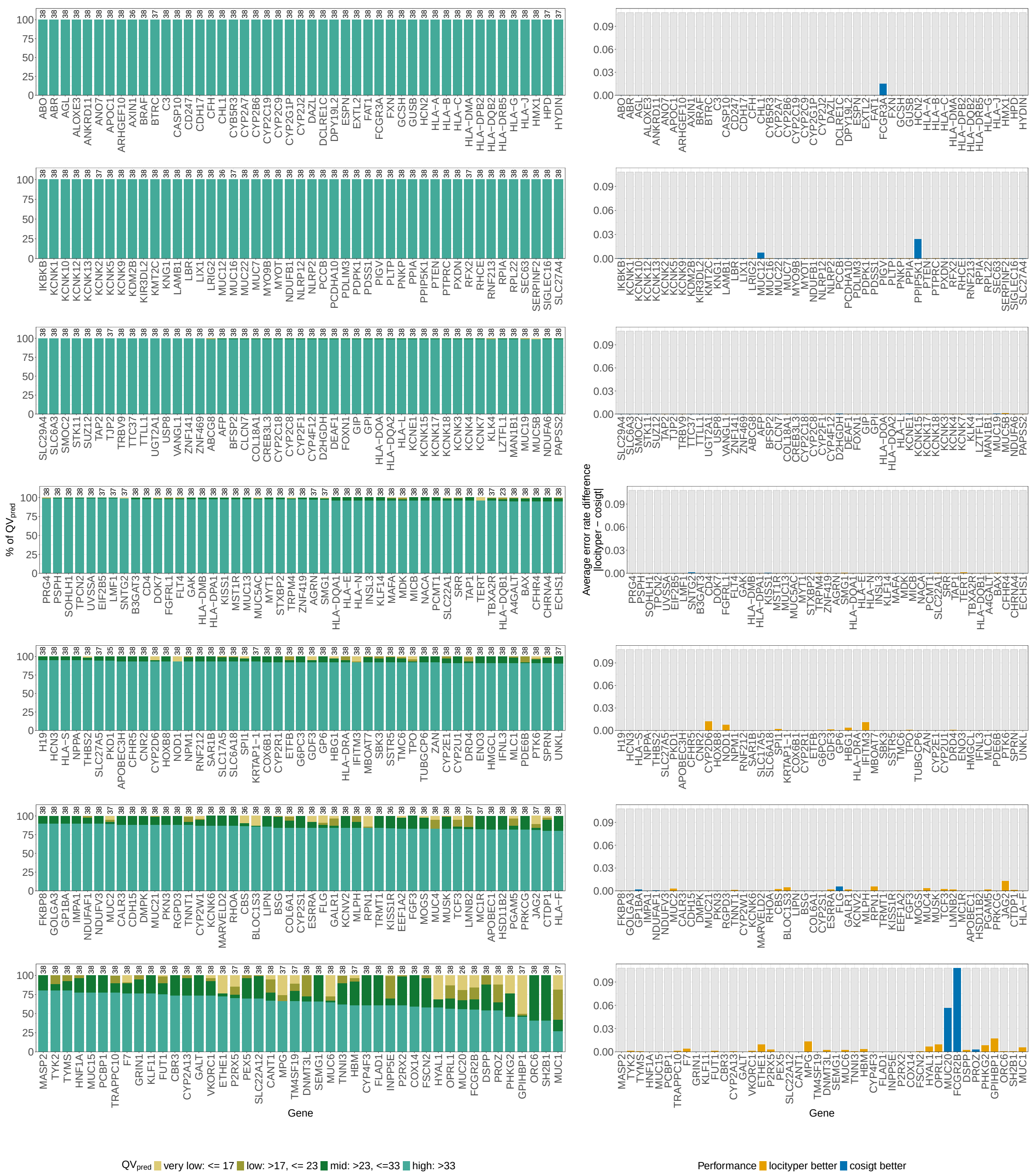

Fig. S6: **Gene-level COSIGT performance in leave-zero-out analysis of CMRGs at 5X coverage.** Per-gene comparison of COSIGT and Locityper genotyping accuracy for 326 CMRGs at 5X coverage. Left: QV_{pred} distribution for COSIGT, showing the percentage of samples in each quality category (very low: <17, low: 17–23, mid: 23–33, high: >33; see Methods). Sample counts per gene are indicated above each bar. Right: Average absolute error rate differences between COSIGT and Locityper. Genes are ordered by COSIGT performance (higher to lower percentage of high QV calls per gene).

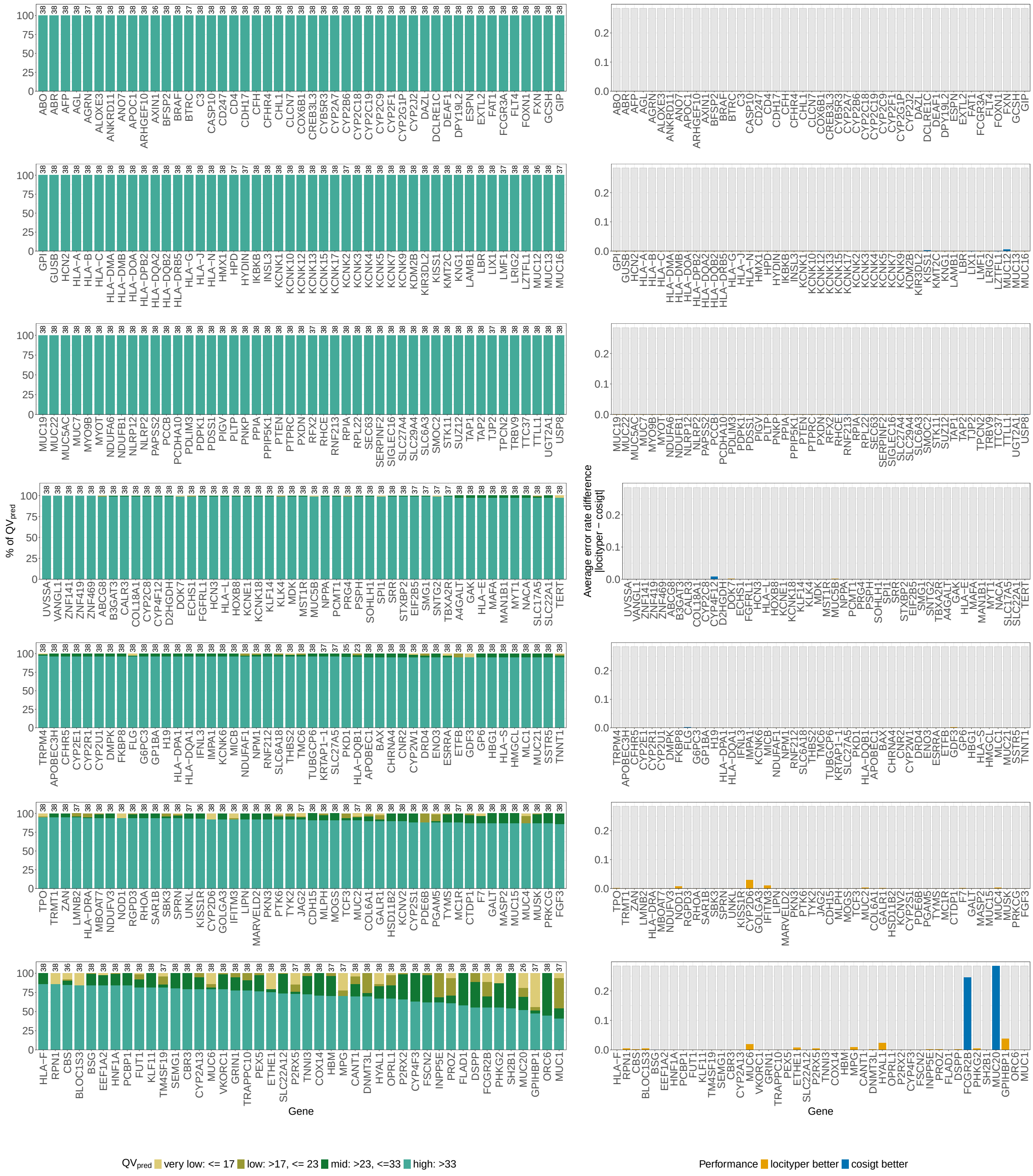

Fig. S7: **Gene-level COSIGT performance in leave-zero-out analysis of CMRGs at 30X coverage.** Per-gene comparison of COSIGT and Locityper genotyping accuracy for 326 CMRGs at 30X coverage. Left: QV_{pred} distribution for COSIGT, showing the percentage of samples in each quality category (very low: <17, low: 17–23, mid: 23–33, high: >33; see Methods). Sample counts per gene are indicated above each bar. Right: Average absolute error rate differences between COSIGT and Locityper. Genes are ordered by COSIGT performance (higher to lower percentage of high QV calls per gene).

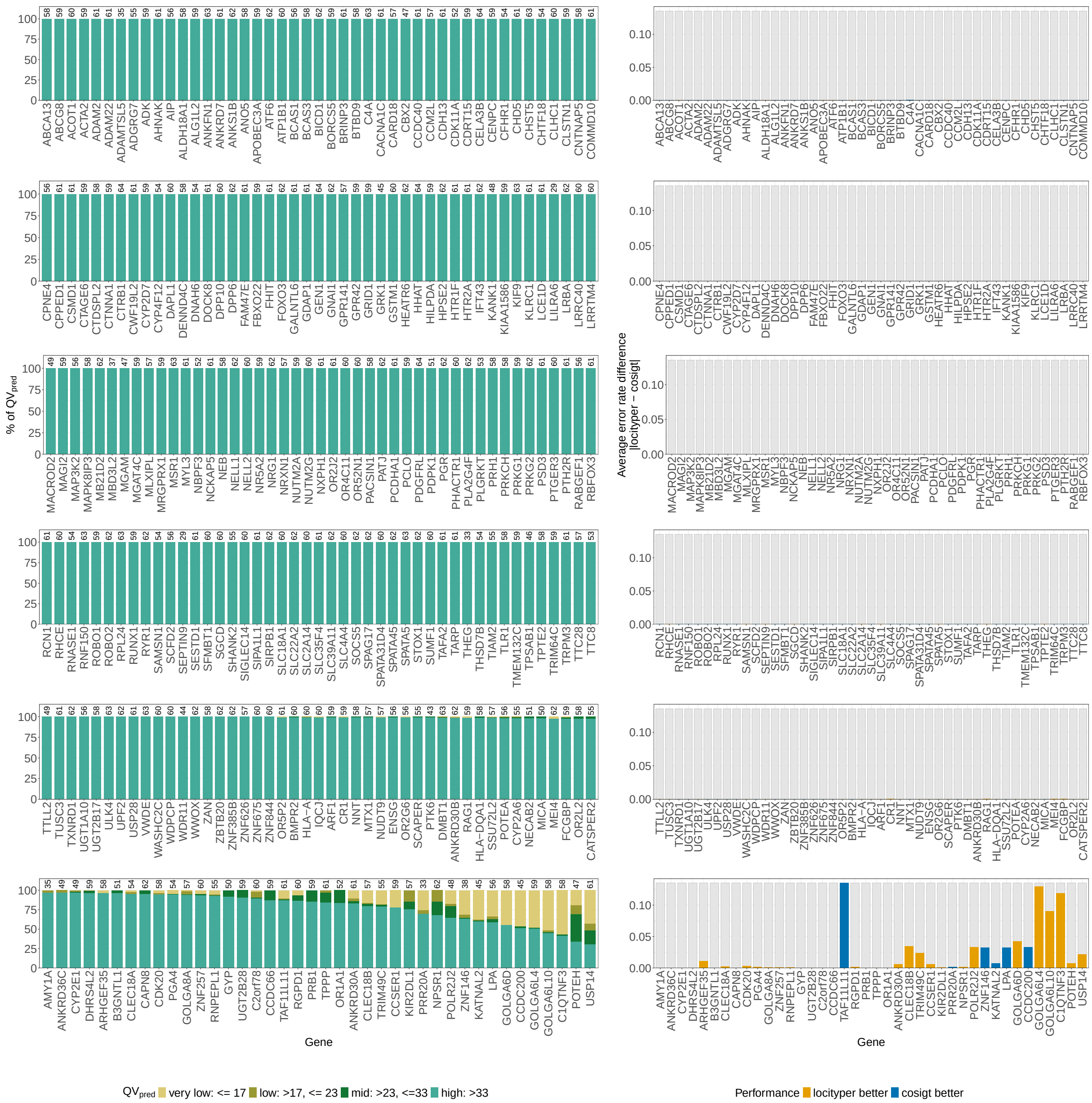

Fig. S8: **Gene-level COSIGT performance in leave-zero-out analysis of SVs at 30X coverage.** Per-gene comparison of COSIGT and Locityper genotyping accuracy for 265 SVs at 30X coverage. Left: QV_{pred} distribution for COSIGT, showing the percentage of samples in each quality category (very low: <17, low: 17–23, mid: 23–33, high: >33; see Methods). Sample counts per gene are indicated above each bar. Right: Average absolute error rate differences between COSIGT and Locityper. Genes are ordered by COSIGT performance (higher to lower percentage of high QV calls per gene).

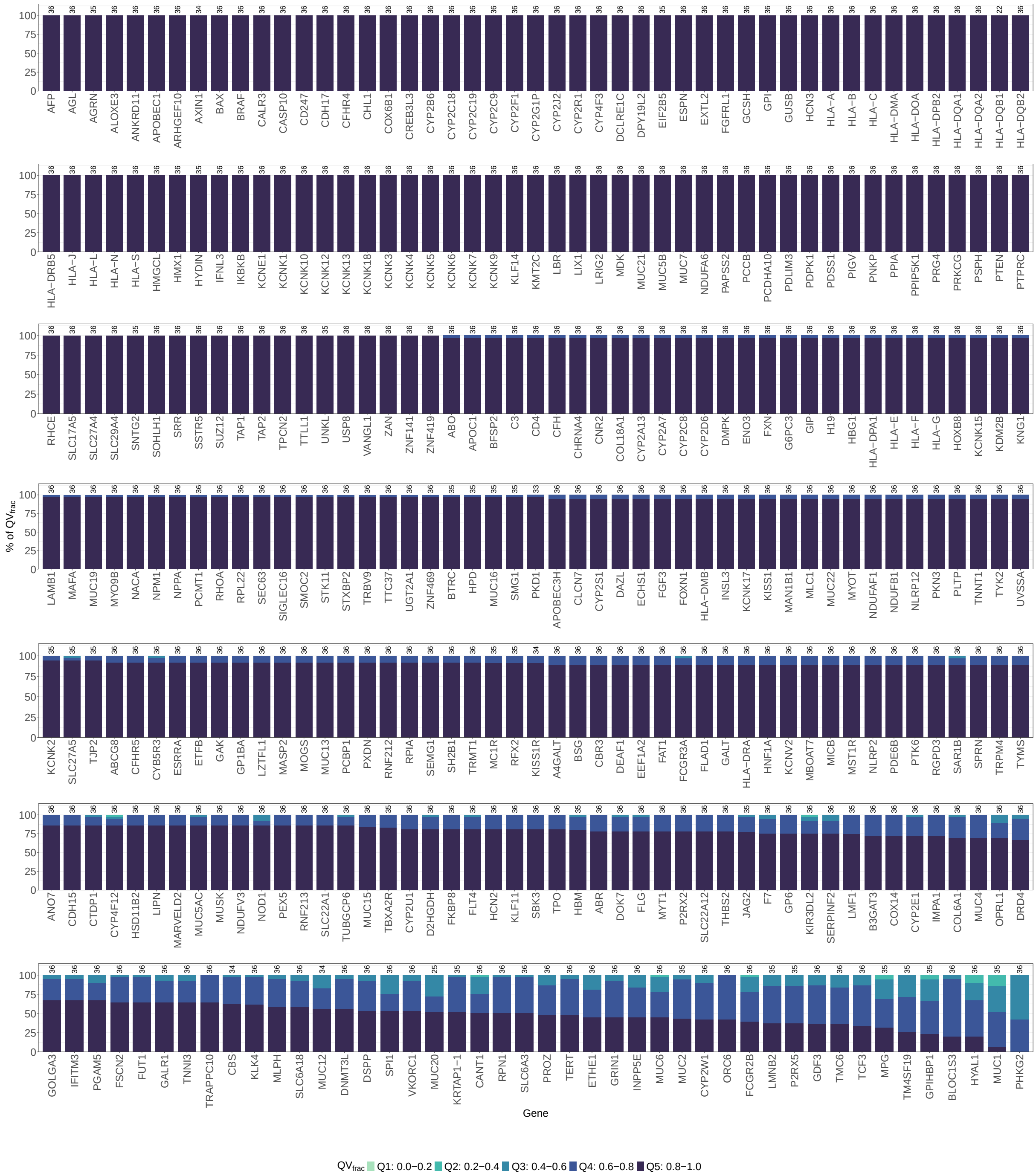

Fig. S9: **Gene-level COSIGT performance in leave-all-out analysis of CMRGs at 30X coverage.** Per-gene distribution of QV_{frac} for 326 CMRGs in the leave-all-out setting. QV_{frac} quantifies how closely COSIGT approaches the best possible accuracy given the available haplotype diversity in the reference panel, with values binned into quintiles (Q1: 0.0–0.2, Q2: 0.2–0.4, Q3: 0.4–0.6, Q4: 0.6–0.8, Q5: 0.8–1.0; see Methods). Genes are ordered by COSIGT performance (higher to lower percentage of Q5 calls per locus). Sample counts per gene are indicated above each bar.

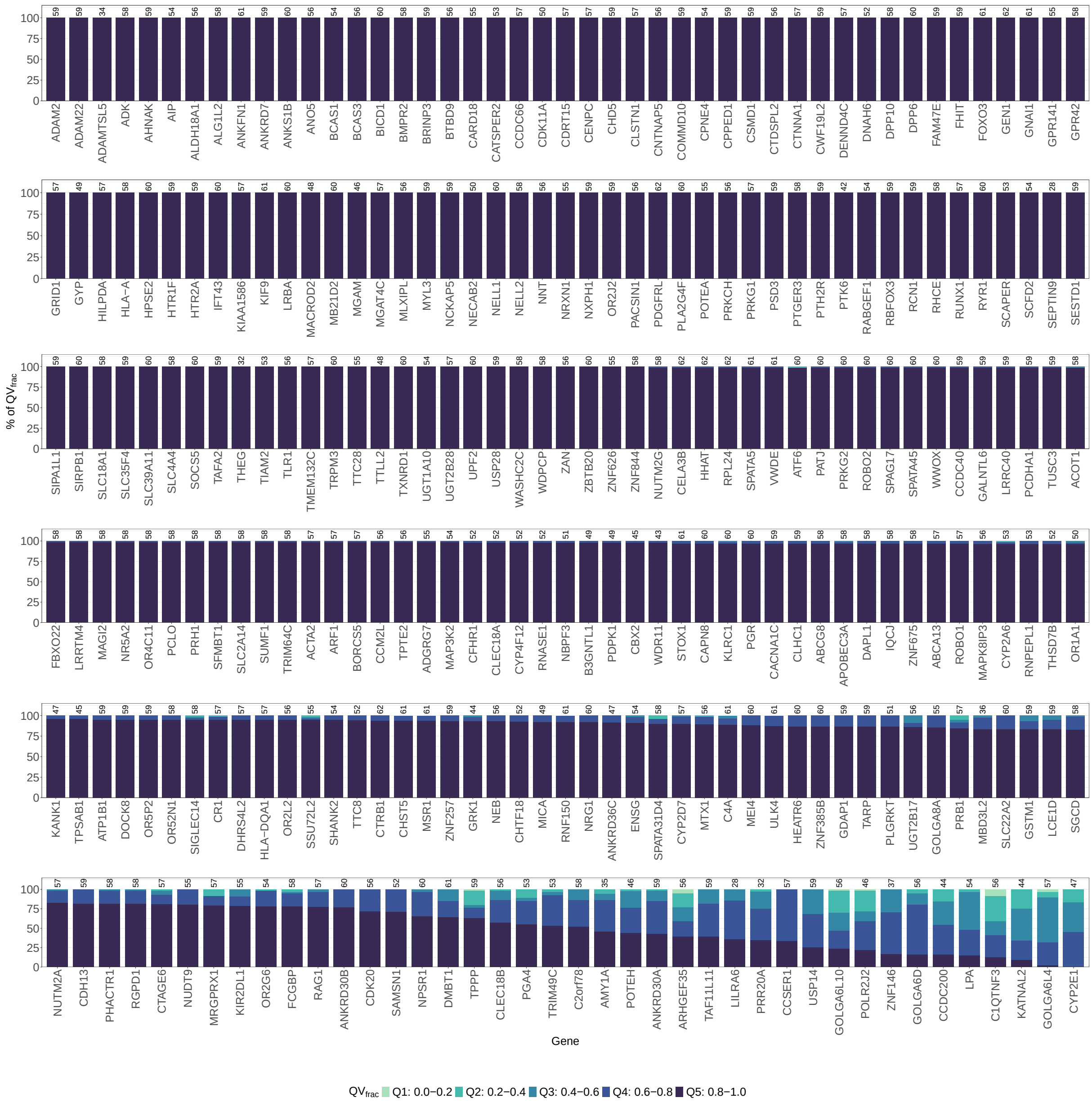

Fig. S10: **Gene-level COSIGT performance in leave-all-out analysis of SVs at 30X coverage.** Per-gene distribution of QV_{frac} for 265 SVs in the leave-all-out setting. QV_{frac} quantifies how closely COSIGT approaches the best possible accuracy given the available haplotype diversity in the reference panel, with values binned into quintiles (Q1: 0.0–0.2, Q2: 0.2–0.4, Q3: 0.4–0.6, Q4: 0.6–0.8, Q5: 0.8–1.0; see Methods). Genes are ordered by COSIGT performance (higher to lower percentage of Q5 calls per gene). Sample counts per gene are indicated above each bar.

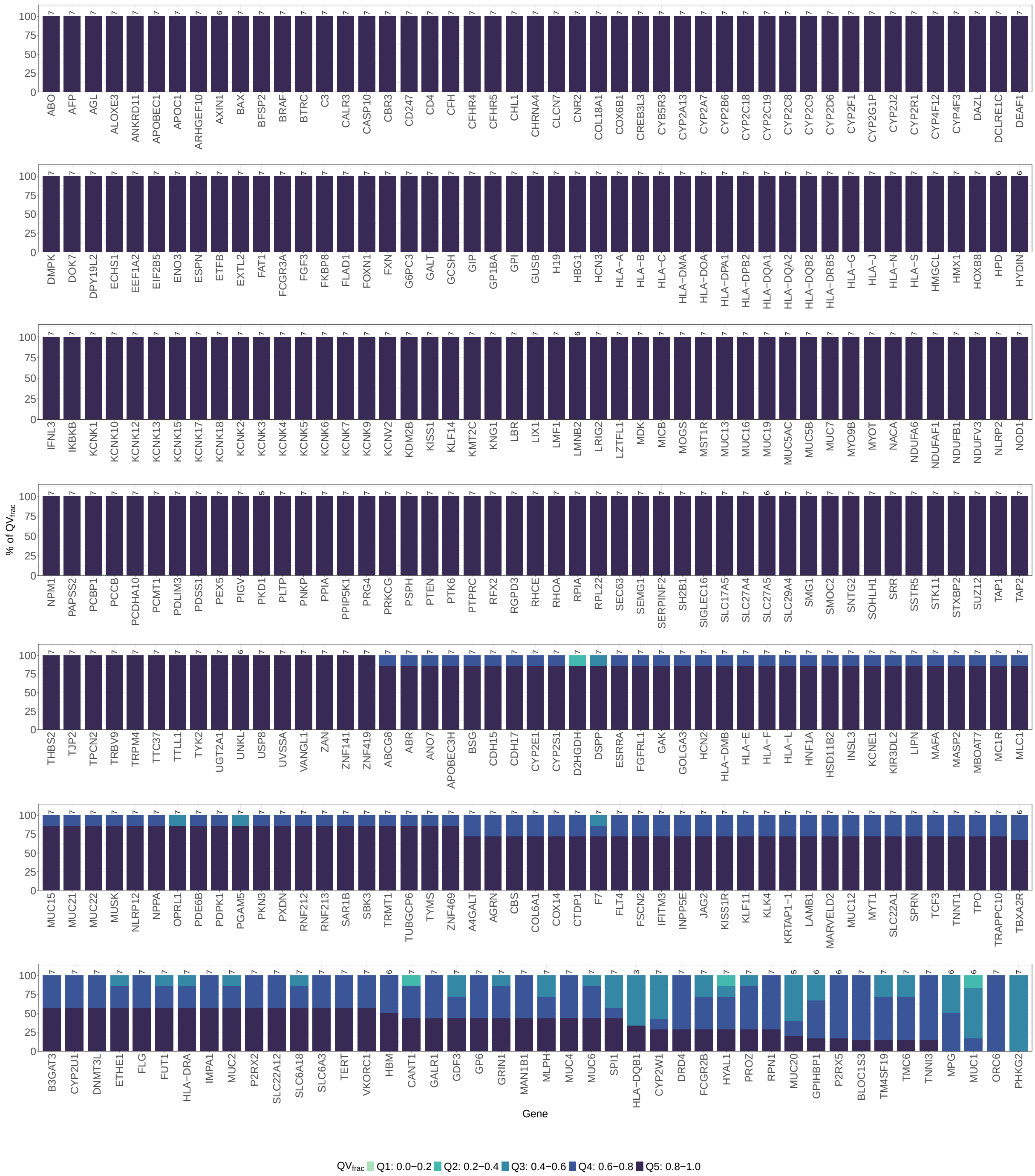

Fig. S11: **Gene-level COSIGT performance in ancestry-mismatched leave-all-out analysis of CMRGs.** Per-gene distribution of QV_{frac} for 326 CMRGs in the ancestry-mismatched leave-all-out setting. QV_{frac} quantifies how closely COSIGT approaches the best possible accuracy given the available haplotype diversity in the reference panel, with values binned into quintiles (Q1: 0.0–0.2, Q2: 0.2–0.4, Q3: 0.4–0.6, Q4: 0.6–0.8, Q5: 0.8–1.0; see Methods). Genes are ordered by COSIGT performance (higher to lower percentage of Q5 calls per locus). Sample counts per gene are indicated above each bar.

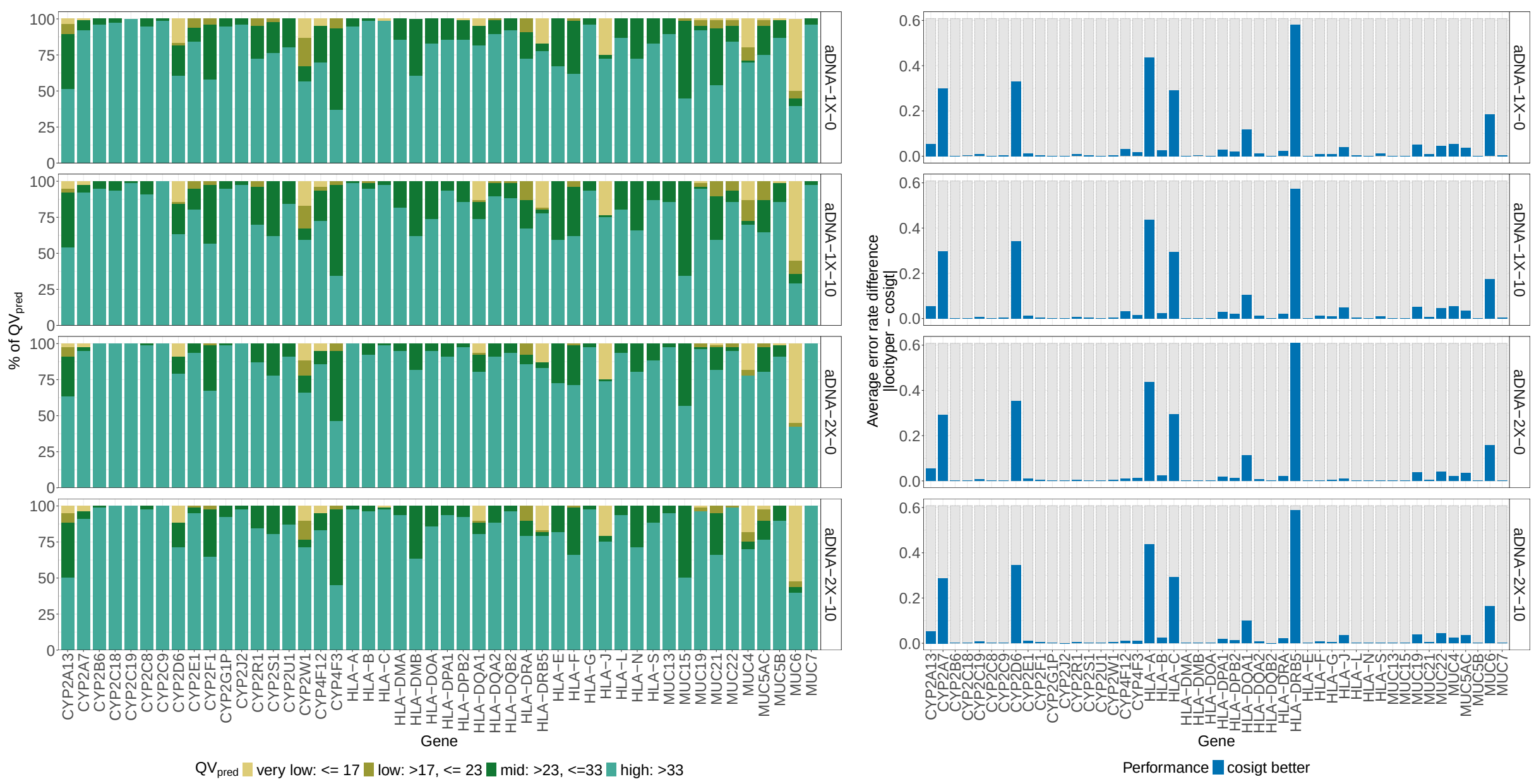

Fig. S12: **Gene-level COSIGT performance on simulated aDNA at variable coverage and contamination.** Per-gene comparison of COSIGT and Locityper genotyping accuracy for 48 selected CMRGs on 38 simulated aDNA samples across four coverage and contamination conditions. Left: QV_{pred} distribution for COSIGT, showing the percentage of samples in each quality category (very low: <17, low: 17–23, mid: 23–33, high: >33; see Methods). Right: Average absolute error rate differences between COSIGT and Locityper. Rows represent different simulated datasets: aDNA at 1X coverage with 0% contamination (aDNA-1X-0), 1X with 10% contamination (aDNA-1X-10), 2X with 0% contamination (aDNA-2X-0), and 2X with 10% contamination (aDNA-2X-10). Genes are ordered alphabetically.

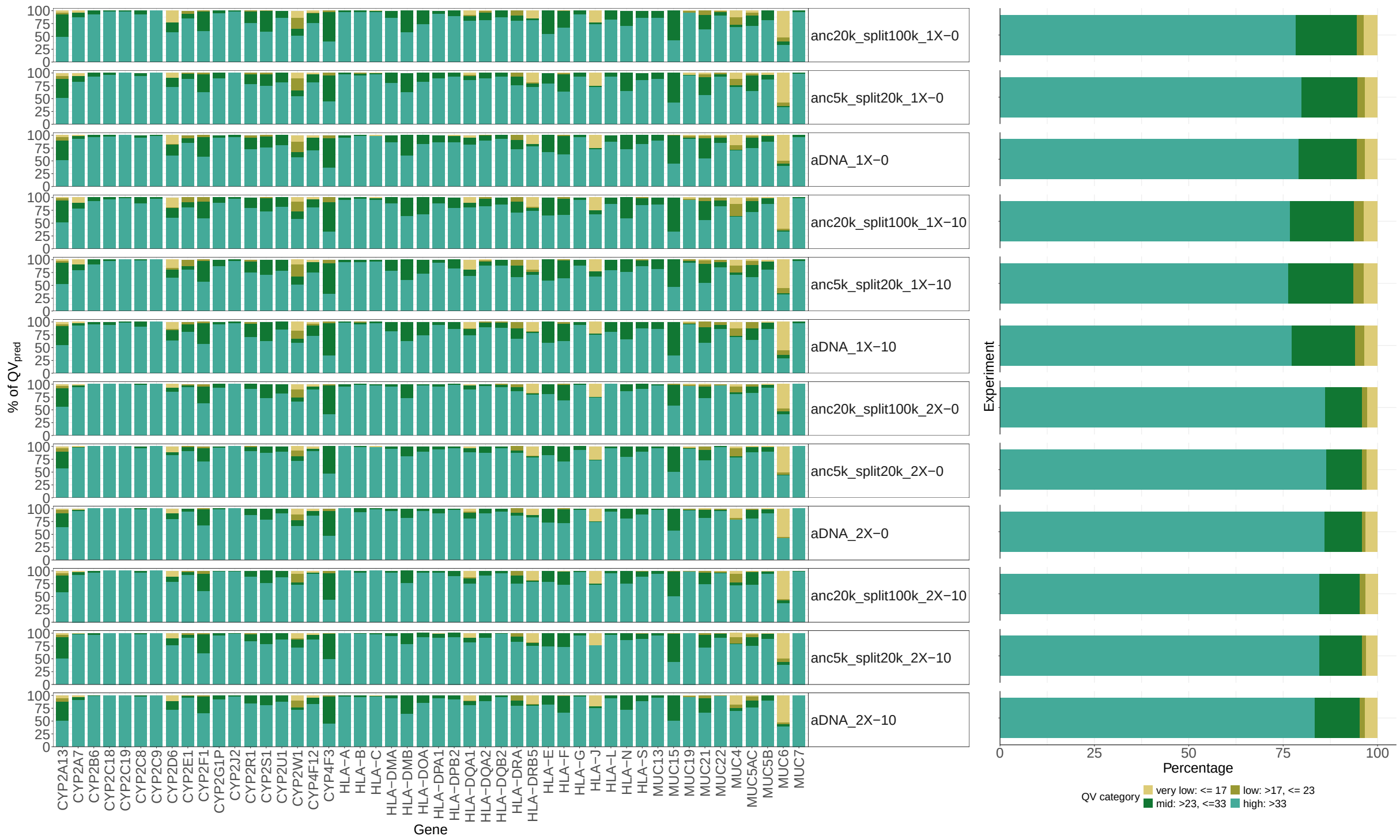

Fig. S13: **Gene-level COSIGT performance on simulated aDNA at variable evolutionary divergence, coverage, and contamination.** Per-gene evaluation of COSIGT genotyping accuracy for 48 selected CMRGs on 38 simulated aDNA samples across 12 evolutionary divergence, coverage, and contamination conditions. Left: QV_{pred} distribution for COSIGT, showing the percentage of samples in each quality category (very low: < 17, low: 17–23, mid: 23–33, high: > 33; see Methods). For each gene, rows represent different simulated datasets across 1X and 2X coverages with 0% and 10% contamination (as in Fig. S12) for three demographic scenarios: standard baseline aDNA, a 5 kya (anc5k) ancient sample with a 20 kya population split (split20k), and a 20 kya ancient sample (anc20k) with a 100 kya split (split100k). Right: Marginal summary panel displaying the global aggregate distribution of quality categories across all 12 simulated conditions combined for each gene. Genes are ordered alphabetically.

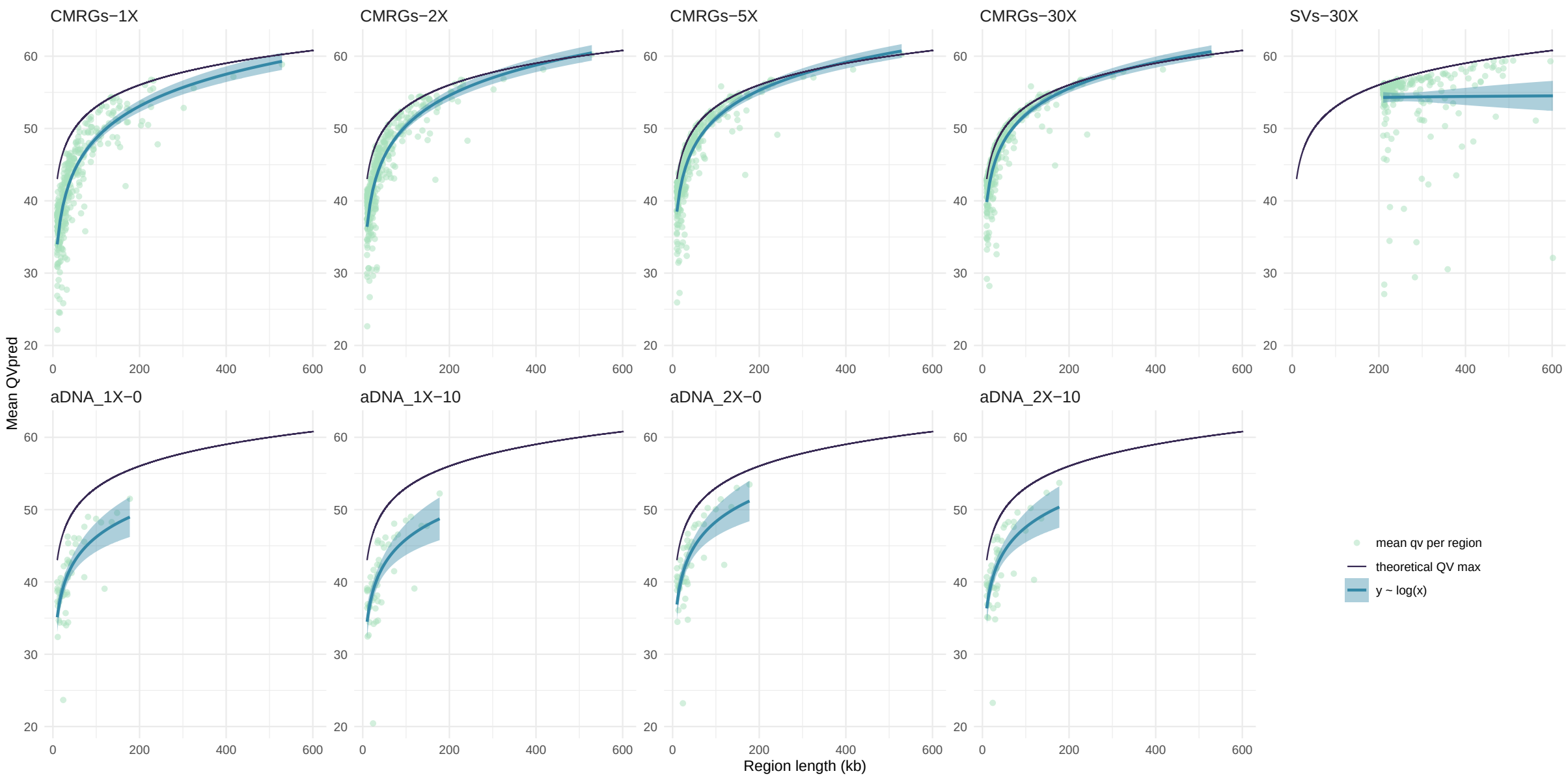

Fig. S14: **COSIGT performance in leave-zero-out analyses stratified by region length.** Scatter plots displaying the relationship between genomic target locus length (kilobases (kb), x-axis) and the mean predicted Quality Value (QV_{pred}, y-axis). Panels represent distinct benchmarking contexts across modern CMRGs, SVs, and simulated aDNA under varying degradation configurations (0% and 10% contamination at 1X and 2X depths). Individual target loci are marked as green points. The solid blue line denotes a logarithmic regression fit ($y = \log(x)$), demonstrating predictable scaling of performance metrics with target footprint size, while the black line indicates the theoretical maximum QV ceiling.

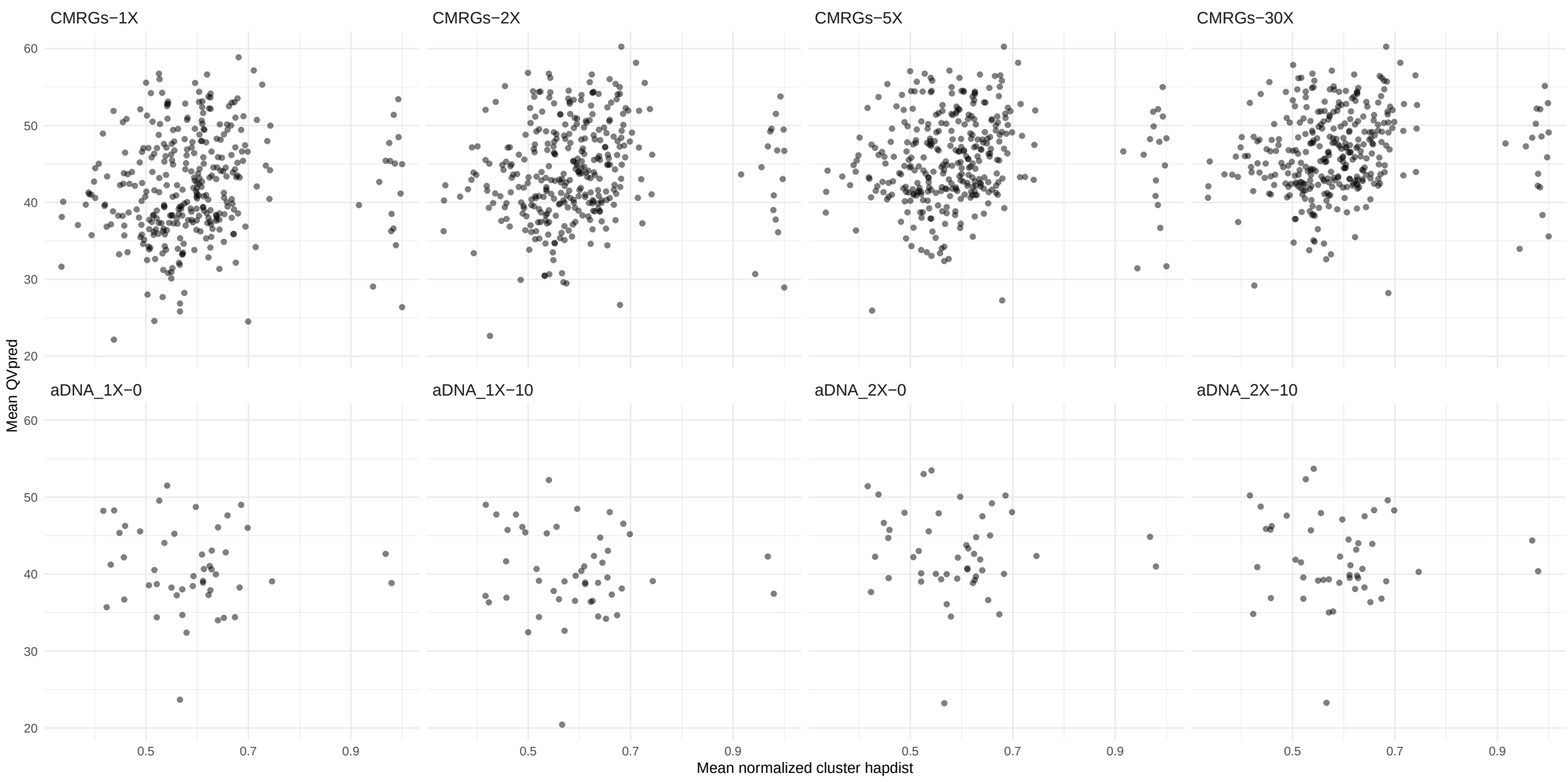

Fig. S15: **COSIGT performance in leave-zero-out analyses stratified by region complexity.** Stratification of mean predicted genotype quality (QV_{pred}, y-axis) against locus complexity, quantified as the mean normalized pairwise cluster distance across all resolved haplotypes embedded within the local variation graph (x-axis). Panels represent distinct benchmarking contexts across modern CMRGs, SVs, and simulated aDNA under varying degradation configurations (0% and 10% contamination at 1X and 2X depths).

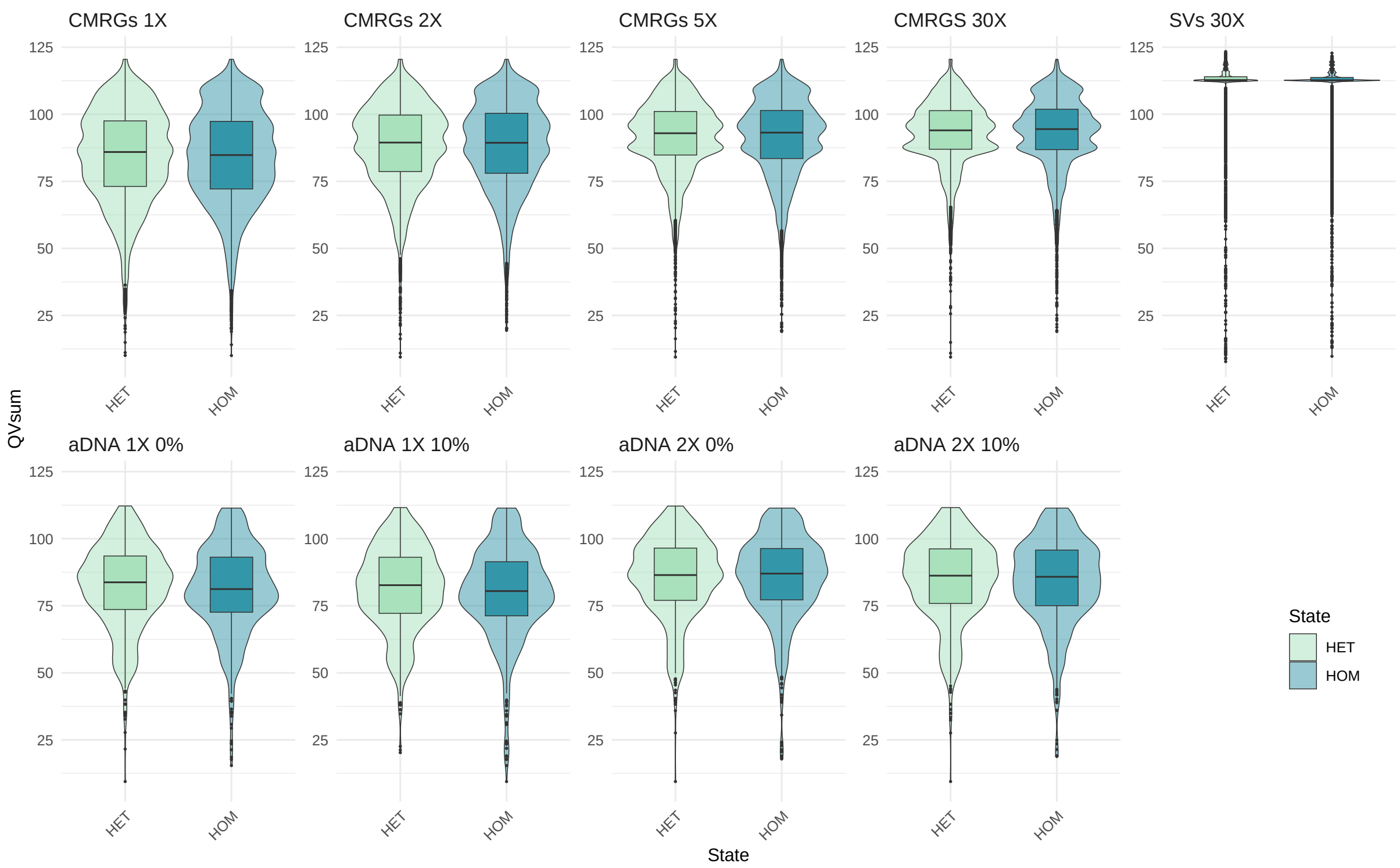

Fig. S16: **COSIGT performance in leave-zero-out analyses stratified by zygosity.** Violin and box plots illustrating the distribution of genotype prediction quality (QV_{sum}, i.e. the sum of QV_{pred} for each sample/region combination) across heterozygous (HET) and homozygous (HOM) states. Zygosity is determined at the haplotype level, where a sample is annotated as homozygous if its two underlying ground-truth haplotypes partition into the identical structural cluster within the reference pangenome graph, and heterozygous otherwise. Panels represent distinct benchmarking contexts across modern CMRGs, SVs, and simulated aDNA under varying degradation configurations (0% and 10% contamination at 1X and 2X depths).

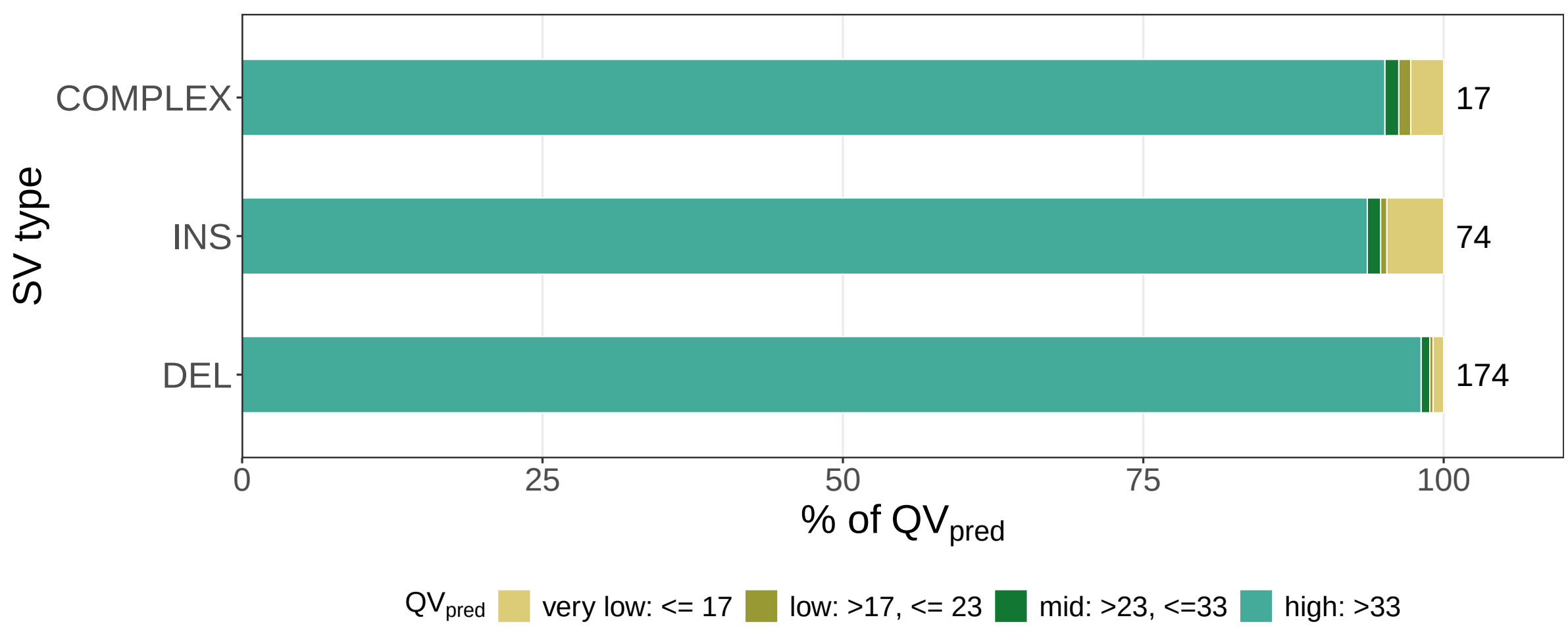

Fig. S17: **COSIGT performance in leave-zero-out analyses stratified by SV type.** Barplots illustrating the distribution of genotype prediction quality (QV_{pred}) for the 265 loci in the SV benchmark. Performance metrics are isolated by distinct canonical SV categories: Deletions (DEL), Insertions (INS), and Complex Events (COMPLEX). SV classification of each locus is dictated strictly by the size of the single largest structural event signature annotated within that target region footprint in the HPRCy1 panel.

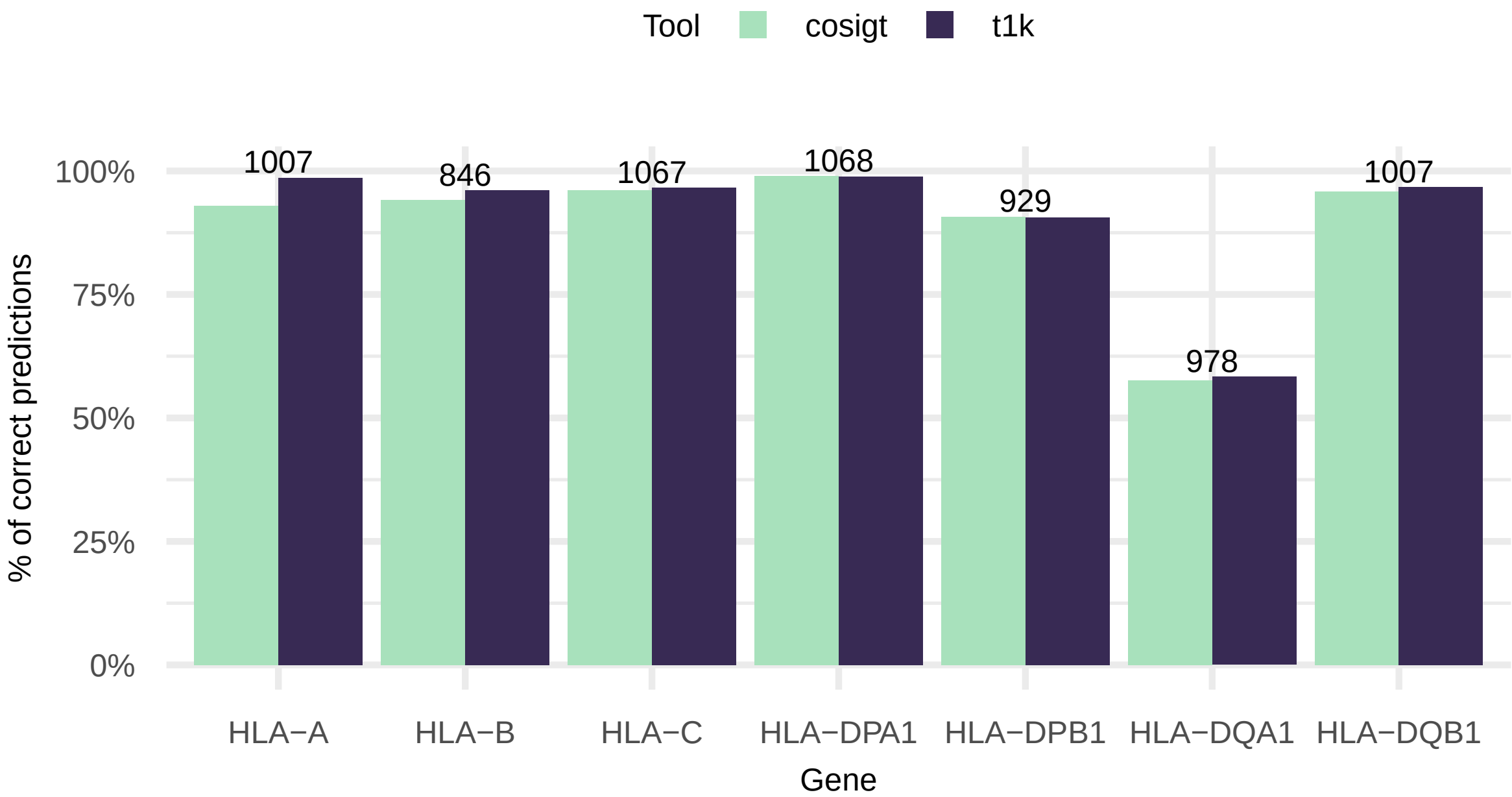

Fig. S18: **COSIGT performance on HLA typing.** Haplotype-level concordance between short-read-based genotyping (COSIGT, T1K) and microarray-based imputation (HLA*IMP:02) for seven HLA genes (*HLA-A, -B, -C, -DPA1, -DPB1, -DQA1, -DQB1*) in a subset ($n = 1{,}085$) of the Moli-sani cohort. Numbers above bars indicate samples with predictions from all three methods and at least one reference haplotype present in the pangenome graph for the imputed genotype. See Methods for details.

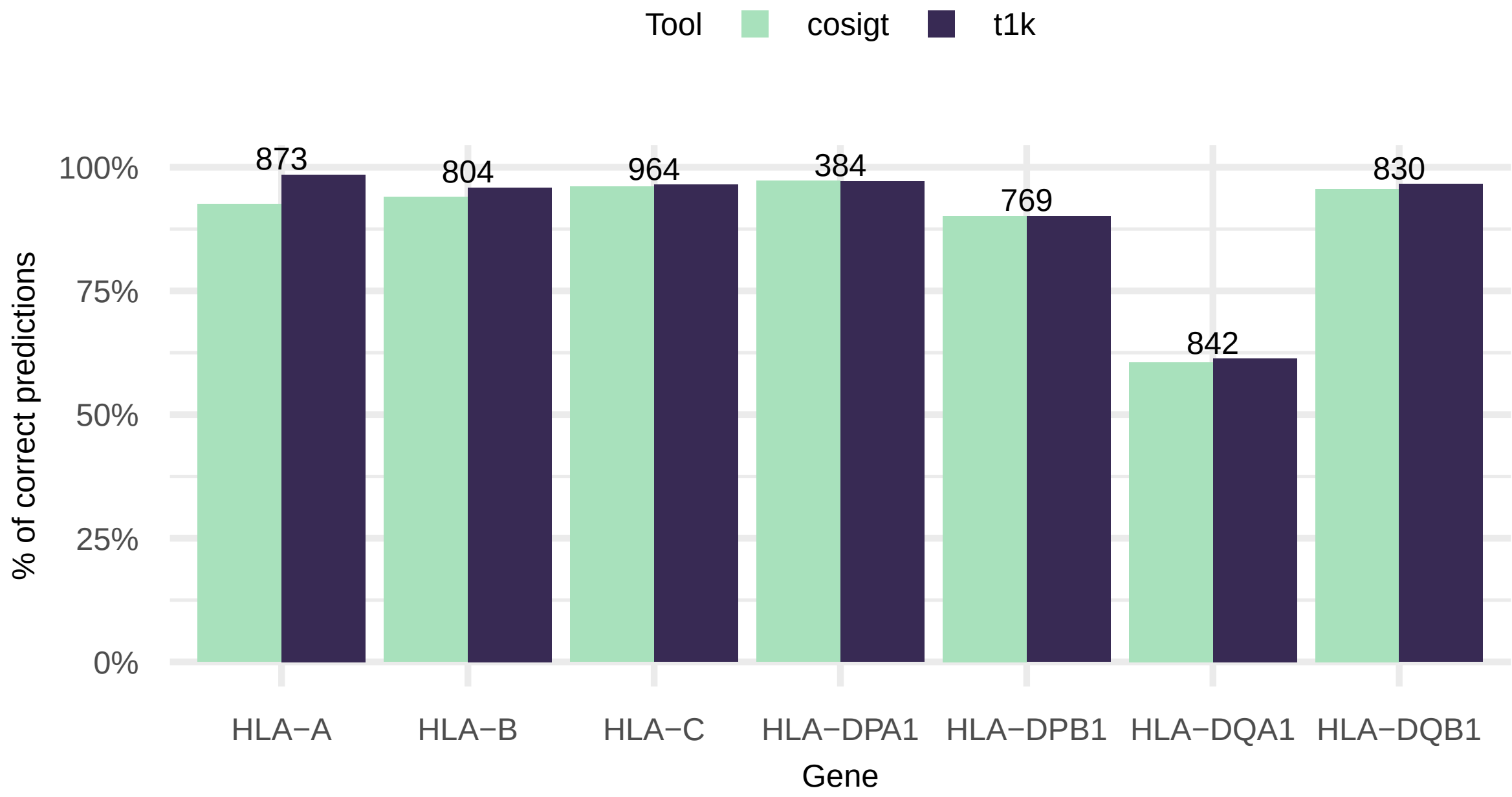

Fig. S19: **COSIGT performance on HLA typing after T1K quality filtering.** Haplotype-level concordance between short-read-based genotyping (COSIGT, T1K) and microarray-based imputation (HLA*IMP:02) for seven HLA genes (*HLA-A, -B, -C, -DPA1, -DPB1, -DQA1, -DQB1*) in a subset (n = 1,085) of the Moli-sani cohort, after filtering for alleles with quality less than or equal to 0 in T1K results. Numbers above bars indicate samples with predictions from all three methods and at least one reference haplotype present in the pangenome graph for the imputed genotype.

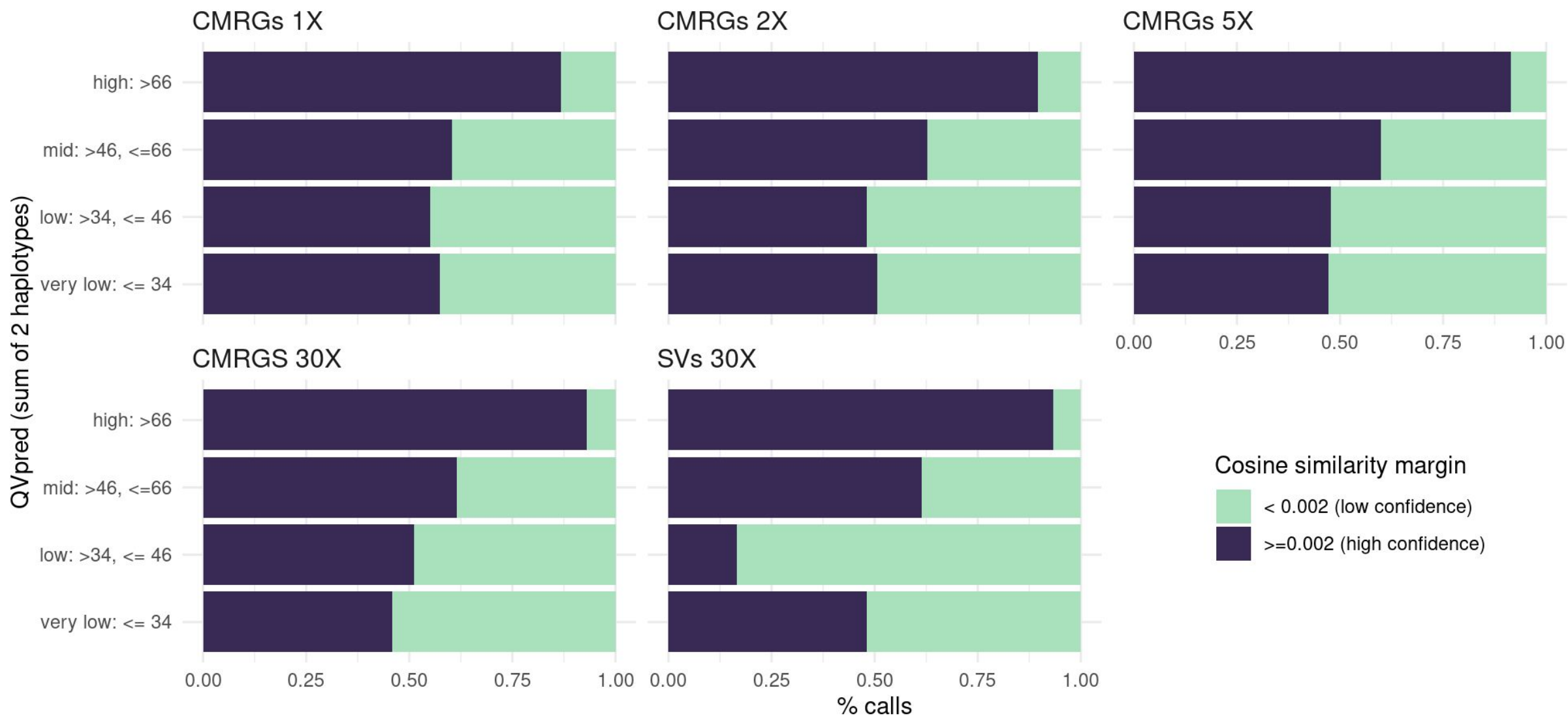

Fig. S20: **Empirical threshold for filtering low-confidence COSIGT genotype calls.** Percentage of predictions for which the cosine similarity margin is above or below the 0.002 threshold, stratified by quality categories (i.e., the sum of QVs for the two predicted haplotypes per sample). The cosine similarity margin is computed as the difference in cosine similarity between the top-ranked haplogroup-pair prediction and the first distinct alternative haplogroup-pair prediction. Calls with narrow margins indicate instances where the top predicted genotype is only weakly separated from alternative genotypes. See **Methods: Genotype inference via cosine similarity**.

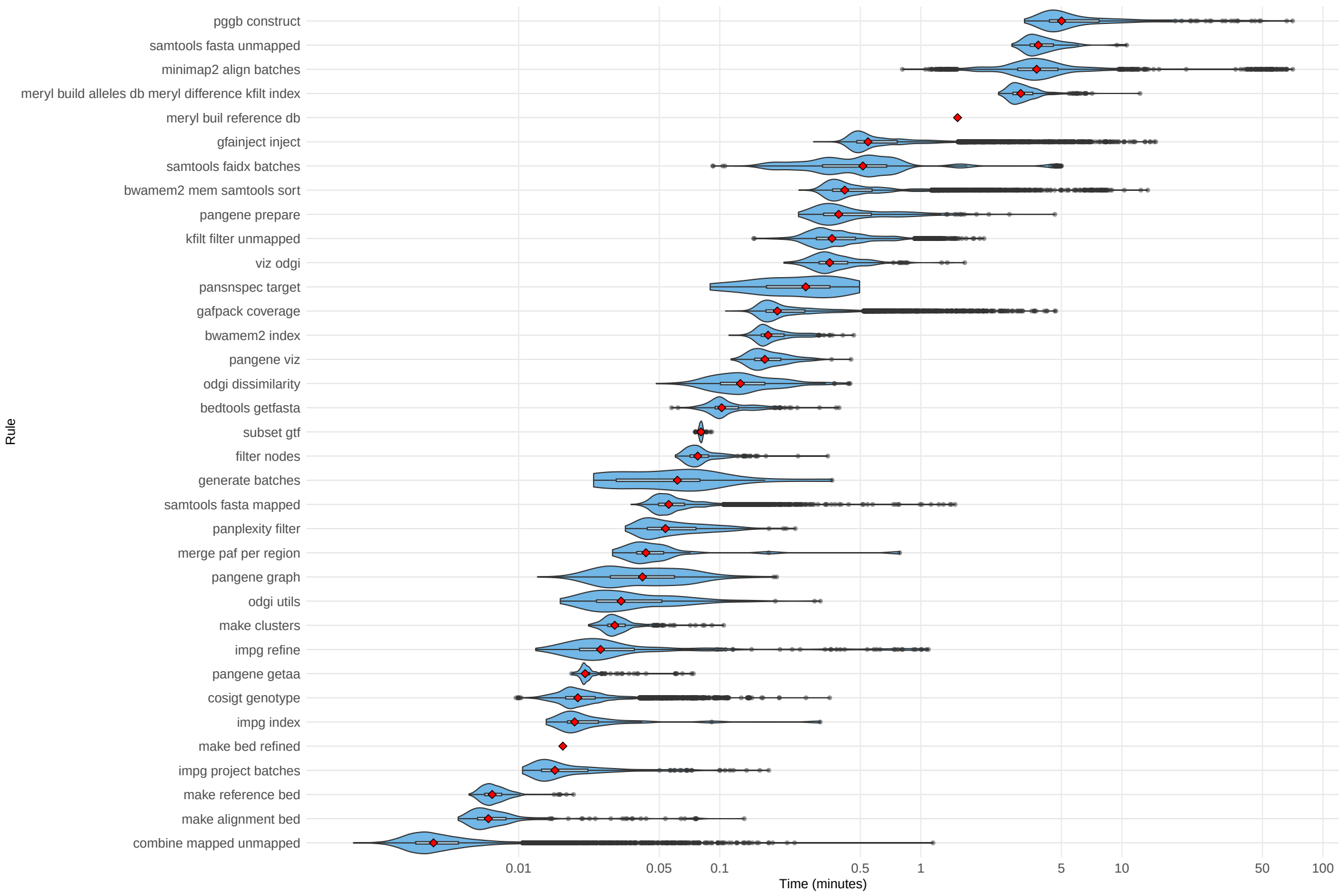

Fig. S21: **COSIGT runtime across pipeline steps.** Violin plots show the distribution of the execution time (minutes) for each pipeline rule across jobs for the leave-zero-out analysis of SVs (see Methods). Boxplots (black) indicate quartiles, and red diamonds mark median values. X-axis uses a logarithmic scale. Rules are ordered by median execution time from slowest (top) to fastest (bottom).

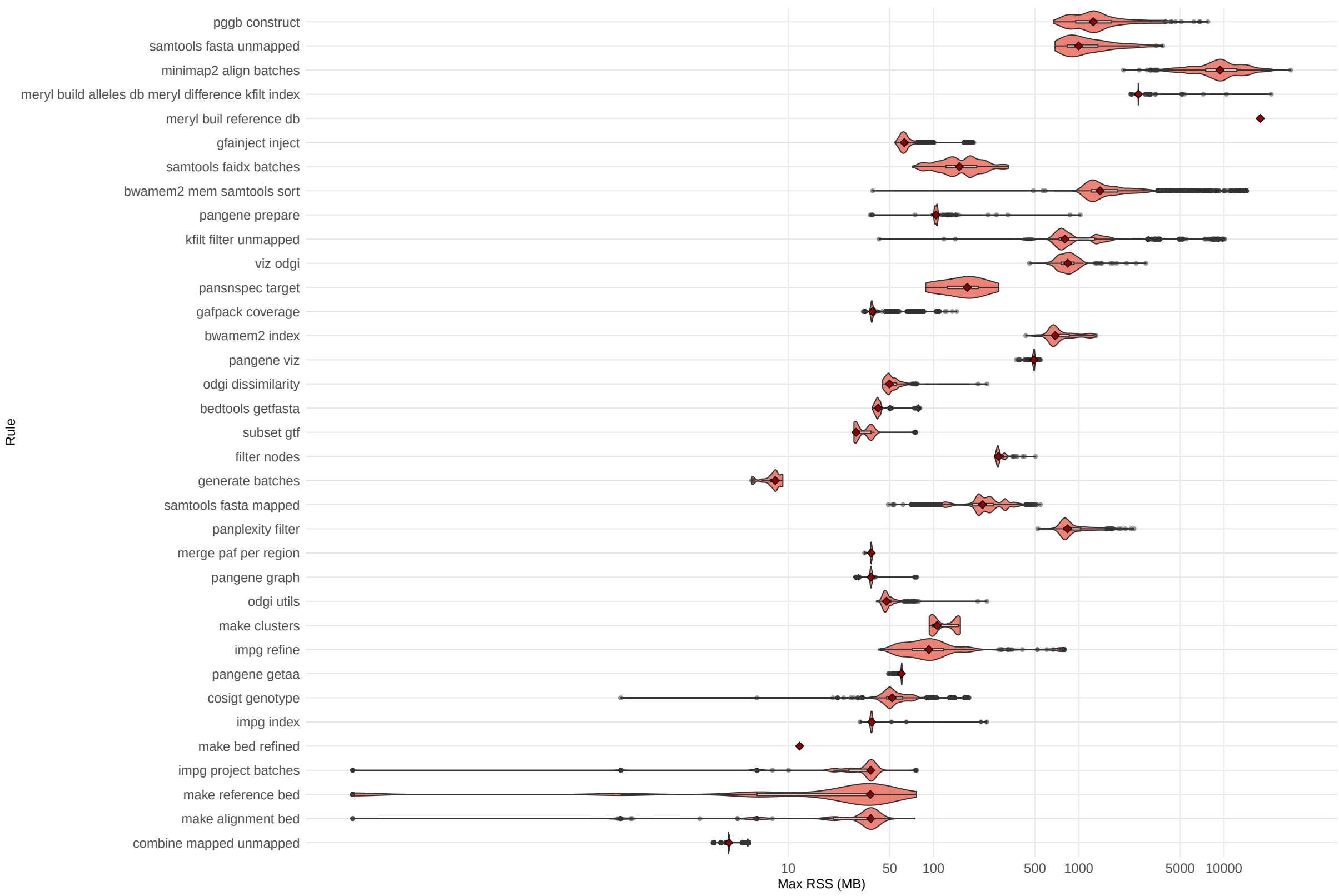

Fig. S22: **COSIGT memory usage across pipeline steps.** Violin plots show the distribution of peak memory consumption (megabytes, MB) for each pipeline rule across jobs for the leave-zero-out analysis of SVs (see Methods). Boxplots (black) indicate quartiles, and red diamonds mark median values. X-axis uses a logarithmic scale. Rules are ordered by median execution time for consistency with Fig. S21.

Supplementary Data Notes

S1. Chromosome partitioning of haplotype-resolved assemblies

The COSIGT pipeline requires haplotype-resolved assemblies partitioned by chromosome to restrict computational analyses to relevant genomic regions. For this study, we used chromosome assignments provided by the HGSVCv3 and HPRCy1 consortia. However, such assignments may not always be available for newly generated assemblies or assemblies from alternative sources.

When reference-based chromosome annotations are unavailable, assemblies can be partitioned using sequence similarity to a reference genome. We provide a reference implementation in the COSIGT documentation (`https://davidebolo1993.github.io/cosigtdoc/usecases/usecases.html`) that uses Mash [1] distance to assign assembly contigs to their most likely chromosome of origin. Briefly, this approach: (1) computes k-mer sketches for both the reference genome and query assembly contigs using Mash, (2) calculates pairwise Mash distances between each contig and reference chromosomes, and (3) assigns each contig to the reference chromosome with the minimum distance.

For each contig, the best-matching reference chromosome is identified by selecting the alignment with the lowest Mash distance. Contigs assigned to the same chromosome are then concatenated into chromosome-specific FASTA files, maintaining the partitioned structure required by the pipeline. The complete workflow, including example commands and parameter settings, is available at `https://davidebolo1993.github.io/cosigtdoc/usecases/usecases.html#option-2-starting-from-individual-haplotypes`.

S2. Incorporating alternative reference contigs for target regions

COSIGT accepts target regions as a standard BED file with genomic coordinates relative to the primary reference assembly. For basic analyses, a three-column BED format (chromosome, start, end) is sufficient. However, two optional columns can be added: column 4 for locus names and column 5 for comma-separated lists of alternative reference contigs corresponding to each target region.

Including alternative contigs is particularly important when working with reference genomes that contain alternate scaffolds, such as the GRCh38 reference used for 1000 Genomes Project alignments. These alternate sequences represent known variation (e.g., MHC haplotypes) and capture reads that would otherwise be unmapped or mismapped to the primary assembly. Failing to include these regions can result in incomplete read extraction and reduced genotyping accuracy, especially for highly polymorphic loci like the HLA genes.

To identify alternative contigs mapping to target regions, we provide a reference workflow in the COSIGT documentation (`https://davidebolo1993.github.io/cosigtdoc/usecases/usecases.html`). The approach: (1) extracts primary chromosomes and alternative scaffolds from the reference genome, (2) aligns alternative scaffolds to primary chromosomes using minimap2, (3) queries target regions against these alignments using impg to identify corresponding alternative contigs, and (4) merges primary and alternative coordinates into an extended BED file.

The resulting BED format enables COSIGT to extract reads mapped to both primary and alternative reference sequences, ensuring comprehensive coverage of sequence diversity at the target locus. For example, HLA typing analyses in this study used extended target regions that included both primary chromosome 6 coordinates and multiple HLA-specific alternative contigs from GRCh38, capturing reads that aligned to any representation of these loci. The

complete workflow with detailed commands is available at `https://davidebolo1993.github.io/cosigtdoc/usecases/usecases.html#incorporating-alternative-contigs`.

Supplementary References

References

[1] Ondov, B.D., Treangen, T.J., Melsted, P., Mallonee, A.B., Bergman, N.H., Koren, S., Phillippy, A.M.: Mash: fast genome and metagenome distance estimation using MinHash. Genome Biology **17**(1), 132 (2016). https://doi.org/10.1186/s13059-016-0997-x
